# Long-Term Results from the IDEAL-CRT Phase 1/2 Trial of Isotoxically Dose-Escalated Radiation Therapy and Concurrent Chemotherapy for Stage II/III Non-small Cell Lung Cancer

**DOI:** 10.1016/j.ijrobp.2019.11.397

**Published:** 2020-03-15

**Authors:** John D. Fenwick, David B. Landau, Angela T. Baker, Andrew T. Bates, Chinnamani Eswar, Angel Garcia-Alonso, Susan V. Harden, Marianne C. Illsley, Virginia Laurence, Zafar Malik, William Philip M. Mayles, Elizabeth Miles, Nazia Mohammed, James Spicer, Paula Wells, Sindu Vivekanandan, Anne-Marie Mullin, Laura Hughes, Laura Farrelly, Yenting Ngai, Nicholas Counsell

**Affiliations:** ∗ Department of Molecular and Clinical Cancer Medicine, Institute of Translational Medicine, University of Liverpool, Liverpool, United Kingdom; † Guy’s & St. Thomas’ NHS Foundation Trust, London, United Kingdom; ‡ Oxford Cancer Centre, Oxford, United Kingdom; § University Hospital Southampton NHS Foundation Trust, Southampton, United Kingdom; ‖ The Clatterbridge Cancer Centre NHS Foundation Trust, Bebington, United Kingdom; # North Wales Cancer Treatment Centre, Rhyl, United Kingdom; ∗∗ Addenbrookes Hospital, Cambridge, United Kingdom; †† Royal Surrey County Hospital NHS Foundation Trust, Guildford, United Kingdom; ‡‡ Poole Hospital NHS Foundation Trust, Poole, United Kingdom; §§ Radiotherapy Trials Quality Assurance Group, Mount Vernon Cancer Centre, Middlesex, United Kingdom; ‖‖ Beatson West of Scotland Cancer Centre, Glasgow, United Kingdom; ∗∗∗ Barts Health NHS Trust, London, United Kingdom; ††† Cancer Research UK & University College London Cancer Trials Centre, London, United Kingdom

## Abstract

**Purpose:**

The IDEAL-CRT phase 1/2 multicenter trial of isotoxically dose-escalated concurrent chemoradiation for stage II/III non-small cell lung cancer investigated two 30-fraction schedules of 5 and 6 weeks’ duration. We report toxicity, tumor response, progression-free survival (PFS), and overall survival (OS) for both schedules, with long-term follow-up for the 6-week schedule.

**Methods and Materials:**

Patients received isotoxically individualized tumor radiation doses of 63 to 71 Gy in 5 weeks or 63 to 73 Gy in 6 weeks, delivered concurrently with 2 cycles of cisplatin and vinorelbine. Eligibility criteria were the same for both schedules.

**Results:**

One-hundred twenty patients (6% stage IIB, 68% IIIA, 26% IIIB, 1% IV) were recruited from 9 UK centers, 118 starting treatment. Median prescribed doses were 64.5 and 67.6 Gy for the 36 and 82 patients treated using the 5- and 6-week schedules. Grade ≥3 pneumonitis and early esophagitis rates were 3.4% and 5.9% overall and similar for each schedule individually. Late grade 2 esophageal toxicity occurred in 11.1% and 17.1% of 5- and 6-week patients. Grade ≥4 adverse events occurred in 17 (20.7%) 6-week patients but no 5-week patients. Four adverse events were grade 5, with 2 considered radiation therapy related. After median follow-up of 51.8 and 26.4 months for the 6- and 5-week schedules, median OS was 41.2 and 22.1 months, respectively, and median PFS was 21.1 and 8.0 months. In exploratory analyses, OS was significantly associated with schedule (hazard ratio [HR], 0.56; 95% confidence interval [CI], 0.32-0.98; *P* = .04) and fractional clinical/internal target volume receiving ≥95% of the prescribed dose (HR, 0.88; 95% CI, 0.77-1.00; *P* = .05). PFS was also significantly associated with schedule (HR, 0.53; 95% CI, 0.33-0.86; *P* = .01).

**Conclusions:**

Toxicity in IDEAL-CRT was acceptable. Survival was promising for 6-week patients and significantly longer than for 5-week patients. Survival might be further lengthened by following the 6-week schedule with an immune agent, motivating further study of such combined optimized treatments.

**Summary**

One-hundred eighteen patients with stage IIB-IV non-small cell lung cancer were treated with isotoxically escalated concurrent chemoradiation to median prescribed doses of 64.5 and 67.6 Gy in 30 fractions over 5 (n = 36) or 6 weeks (n = 82). Toxicity was acceptable for both schedules. Overall survival was longer in 6-week than in 5-week patients (median, 41.2 vs 22.1 months; hazard ratio, 0.56; 95% confidence interval, 0.32-0.98; *P* = .04). Progression-free survival was also longer in 6-week patients (median, 21.1 vs 8.0 months; hazard ratio, 0.53; 95% confidence interval, 0.33-0.86; *P* = .01).

## Introduction

Dose-escalated concurrent chemoradiation (CRT) should be a good treatment for non-small cell lung cancer (NSCLC), since a dose-response has been demonstrated for local progression-free survival (PFS) rates after radiation therapy (RT) for NSCLC,^1^ and a hazard ratio (HR) of 0.84 (*P* = .04) has been reported for mortality in concurrent versus sequential arms of randomized trials of CRT.^2^ A recent meta-analysis, however, found a median survival ratio of 0.83 (*P* = .02) for dose-escalated versus conventional concurrent CRT.^3^

This subunity survival ratio was driven largely by results from the Radiation Therapy Oncology Group (RTOG)-0617 phase 3 study of concurrent CRT, which trialed 60 and 74 Gy radiation doses in 30 and 37 fractions of 2 Gy over 40 and 51 days, respectively, and found significantly lower survival for the high-dose arm.^4^ Dose prescription in RTOG-0617 was to 95% of the planning target volume (PTV), leading to isocenter doses roughly 5% higher than prescribed levels,^5^^,^^6^ corresponding to 63 and 78 Gy in 2.1 Gy fractions.

Here we report mature results from the multicenter phase 1/2 IDEAL-CRT trial of individually dose-escalated concurrent CRT for stage IIB/III NSCLC.^7^ IDEAL-CRT used isotoxic dose prescription to limit toxicity^8^ and dose-per-fraction escalation to avoid schedule protraction.^9^ Two 30-fraction schedules were investigated, one after the other, using the same eligibility criteria for patient recruitment to both.

Tumor isocenter doses of 63 to 73 Gy were given over 40 days in the first schedule (6 weeks, 5 fractions per week), whereas 63 to 71 Gy was given over 33 days in the second schedule (5 weeks, 6 fractions per week, including 2 on the same day separated by a minimum 6 hour gap). We have previously reported early survival and toxicity data for the 6-week schedule,^7^ and now present corresponding results for the 5-week schedule with longer-term follow-up data for the 6-week schedule.

## Methods and Materials

Patients enrolled on this nonrandomized study received RT concurrent with 2 cycles of cisplatin and vinorelbine. Inclusion criteria were histologically/cytologically confirmed stage IIA-IIIB NSCLC, World Health Organization performance status (PS) 0 or 1, suitability for CRT agreed by multidisciplinary team, no prior anticancer therapy, forced expiratory volume ≥1 L or 40% of predicted, carbon monoxide diffusing capacity ≥40% predicted, biochemistry and hematology baselines suitable for chemotherapy, and glomerular filtration rate ≥60 mL/min. Exclusion criteria were chronic liver disease or bilirubin >35 μmol/L, connective tissue disorders, and history of prior malignancy likely to interfere with the protocol treatment.

### Design

Tumor doses were prescribed to the highest levels achievable while meeting the normal tissue and target coverage dose-limits shown in Table E1 (available online at https://doi.org/10.1016/j.ijrobp.2019.11.397). Consequently the prescribed doses differed according to tumor size and proximity to normal structures, the dose-limits set for the 2 schedules, and potentially RT technique and planning. Lung, spinal cord, brachial plexus, and heart dose limits were set at levels determined in an earlier review.^7^^,^^10^ To limit central blood vessel and airway damage, ceilings of 71 and 73 Gy were placed on prescribed tumor doses delivered using the 5- and 6-week schedules,^10^ the 5-week ceiling being slightly lower to allow for possible effects of treating twice on 1 day each week.^11^ Cord and brachial plexus limits were kept the same for both schedules because the 6-week schedule limits were considered conservative.

Insufficient data existed to define an esophageal constraint ahead of IDEAL-CRT, and therefore increasing experimental constraints on the maximum dose deliverable to 1 cm^3^ of esophagus were trialed sequentially.^7^ Patients were split into 2 nonrandomized groups based on dosimetric findings: Group 1, in which prescribed tumor doses were limited by the experimental esophageal constraints (65, 68, and 71 Gy for the 6-week schedule, 65 Gy alone for the 5-week schedule), and Group 2, in which prescribed doses were limited by other constraints, often lung or spinal cord. A 63 Gy constraint was initially placed on the maximum dose to 1 cm^3^ of esophagus for Group 2 patients and was raised when results from Group 1 showed safety for early esophageal toxicity at higher dose-levels.

Planned recruitment for phase 1 testing of the 5-week schedule was 12 patients to Group 1, each receiving 65 Gy to 1 cm^3^ of esophagus, and ≥23 patients to Group 2 to provide additional toxicity data and to reject a grade 3 to 5 pneumonitis rate of ≥20%, with 80% power and 10% 1-sided significance level (using a single sample test for proportions), assuming an underlying rate of ≤7%. For Group 1 of the 6-week schedule, 6 or 12 patients each were recruited at the 65, 68, and 71 Gy esophageal limits following a phase 1, 6 + 6 design.^7^

In a phase 2 element of IDEAL-CRT, survival and toxicity data have been analyzed jointly across Groups 1 and 2 of the 5- and 6-week schedules.

### Procedures

RT planning was carried out using 3-dimensional (3D) or 4-dimensional (4D) computed tomography (CT) images collected during quiet breathing. On 3D-CT, the gross tumor volume (GTV) was contoured and expanded by 5 mm to form a clinical target volume (CTV), and by an additional 5 mm minimum radially and 10 mm minimum craniocaudally to create a PTV.^12^ On 4D-CT, GTVs were outlined on individual scan phases and merged to form a composite volume, which was expanded by 5 mm to form an internal target volume (ITV) including microscopic spread, and by a further 5 mm minimum to form a PTV.^12^ Treatments used 5 to 8 MV photon beams and volumetric modulated arc therapy, or 3- to 5-field conformal techniques. Dose distributions were calculated using “type-b” superposition-convolution algorithms,^13^ and tumor doses were prescribed to the International Commission on Radiation Units reference point. Quality assurance processes have previously been described.^7^

Individualized prescribed tumor doses were initially selected to achieve a value of 18.2 Gy for each patient’s lung EQD2_mean_, the equivalent dose in 2 Gy fractions averaged over both lungs excluding the GTV.^14^^,^^15^ They were then reduced by 10% and further modified as detailed in Table E1 (available online at https://doi.org/10.1016/j.ijrobp.2019.11.397) to avoid tumor de-escalation or exceeding normal tissue dose constraints.

Concurrent chemotherapy comprised 2 cycles, giving 75 mg/m^2^ cisplatin intravenously on day 1 of RT and 15 mg/m^2^ vinorelbine intravenously on days 1 and 8 (cycle 1), and the same doses on days 22 and 29 of the 5-week schedule and days 29 and 36 of the 6-week schedule (cycle 2).

Positron emission tomography scanning and staging CT of the thorax and abdomen were performed for all patients, followed within 42 days of the later scan by planning CT. During RT, weight, PS, dyspnea score, and full blood count were clinically assessed weekly. After RT, weight, PS, dyspnea score, pulmonary status, adverse events (AEs), and toxicity data were collected at clinical reviews held weekly during the first month, then monthly to 6 months, 3 monthly to 24 months, 6 monthly to 36 months, and annually thereafter. Lung function tests and CT of the thorax and abdomen were carried out 3, 6, 12, and 24 months after RT. Chest radiographs were taken at 1, 3, 12, 18, and 24 months. Electrocardiograms were collected at baseline and 6 and 12 months post-RT.

### Outcomes and statistics

All patients who received at least 1 RT fraction were included in this analysis of long-term trial data. Endpoints for each schedule were overall survival (OS), PFS, tumor response (Response Evaluation Criteria in Solid Tumors version 1.1), and toxicity, whose attribution to treatment was overseen by an independent data monitoring committee. Esophagitis, pneumonitis, and other pulmonary toxicities were graded according to Common Terminology Criteria for Adverse Events (version 4.0), and nonpulmonary late radiation toxicities according to the RTOG/European Organisation for Research and Treatment of Cancer late radiation morbidity scoring system.

Time-to-event endpoints were measured from the start of treatment, censored at the date last seen, and estimated using the Kaplan-Meier method to allow for each patient’s length of follow-up. Exploratory univariable and multivariable analyses of associations between these endpoints and patient- and treatment-related factors were performed using Cox proportional hazards regression. Stepwise bidirectional elimination, including all factors with *P* < .20, was used to find the model with the lowest Akaike Information Criterion score. Reported confidence intervals (CIs) and significance levels are 2-sided except where otherwise indicated.

### Role of the funding source

Trial conduct and analysis were the responsibility of the sponsor, University College London. The funder, Cancer Research UK, was not involved in trial conduct, analysis or interpretation, or reporting of results. The corresponding author had full access to the study data and final responsibility to submit for publication. The trial was run in accordance with the Declaration of Helsinki and with the approval of all relevant ethical bodies and regulatory authorities.

## Results

In total, 120 patients were recruited to IDEAL-CRT: 84 to the 6-week schedule from 8 UK centers between October 2010 and March 2013, and 36 (12 Group 1, 24 Group 2) to the 5-week schedule, 34 from the original 8 centers and 2 from an additional UK center, between May 2013 and March 2015.

Patient characteristics and treatment details are shown in Tables 1 and 2. Overall, median patient age was 66 years (range, 43-84 years), median GTV size was 101 cm^3^ (range, 14-602 cm^3^), and 29% of patients were female. Forty-three percent of patients were PS 0 and 57% were PS 1. Fifty-eight percent of patients had squamous tumor histology, 28% adenocarcinoma, and 14% other. Disease stage was IIB, IIIA, IIIB, and IV in 6%, 68%, 26%, and 1% of patients, respectively.

**Table 1:** Patient baseline characteristics

	6-week schedule		5-week schedule		Total
	Group 1 (n = 35)	Group 2 (n = 49)	Group 1 (n = 12)	Group 2 (n = 24)	(n = 120)
Age, median (range), y	66 (46-84)	66 (43-78)	62 (46-74)	68 (61-76)	66 (43-84)
Sex					
Female	9 (26%)	13 (27%)	5 (42%)	8 (33%)	35 (29%)
Male	26 (74%)	36 (73%)	7 (58%)	16 (67%)	85 (71%)
WHO PS					
0	12 (34%)	20 (41%)	5 (42%)	15 (63%)	52 (43%)
1	23 (66%)	29 (59%)	7 (58%)	9 (37%)	68 (57%)
MRC dyspnea score∗					
0	10 (31%)	15 (33%)	6 (50%)	10 (42%)	41 (36%)
1	12 (38%)	22 (48%)	5 (42%)	12 (50%)	51 (45%)
2	8 (25%)	6 (13%)	1 (8%)	1 (4%)	16 (14%)
3	2 (6%)	3 (7%)	0	1 (4%)	6 (5%)
Histology					
Adenocarcinoma	12 (34%)	14 (29%)	2 (17%)	5 (21%)	33 (28%)
Squamous	17 (49%)	30 (61%)	8 (67%)	15 (63%)	70 (58%)
Other NSCLC	6 (17%)	5 (10%)	2 (17%)	4 (17%)	17 (14%)
Stage (UICC TNM, seventh edition)					
IIA	0	0	0	0	0
IIB	0	6 (12%)	0	1 (4%)	7 (6%)
IIIA	24 (69%)	33 (67%)	8 (67%)	16 (67%)	81 (68%)
IIIB	11 (31%)	10 (20%)	4 (33%)	6 (25%)	31 (26%)
IV	0	0	0	1 (4%)	1 (1%)
GTV size, median (range), cm^3^	110 (14-602)	94 (15-329)	46 (21-164)	105 (21-217)	101 (14-602)

*Abbreviations:* GTV = gross tumor volume; MRC = Medical Research Council; NSCLC = non-small cell lung cancer; UICC = Union Internationale Contre le Cancer; WHO PS = World Health Organization performance status.

∗ Baseline dyspnea scores available for 78 of 84 patients treated using the 6-week schedule.

**Table 2:** Patient treatment details

	6-week schedule		5-week schedule		Total
	Group 1 (n = 35)	Group 2 (n = 47)	Group 1 (n = 12)	Group 2 (n = 24)	(n = 118∗)
Prescribed dose, median (range), Gy	69.0 (63.0-73.0)	65.5 (63.0-73.0)	64.8 (63.4-68.4)	63.6 (63.0-71.0)	66.0 (63.0-73.0)
RT duration,† median (range), d	40 (38-44)	40 (5-47)	33 (33-38)	33 (33-35)	40 (5-47)
Technique					
3D conformal	34 (97%)	45 (96%)	9 (75%)	16 (67%)	104 (88%)
VMAT	1 (3%)	2 (4%)	3 (25%)	8 (33%)	14 (12%)
Planning CT					
3D	18 (51%)	29 (62%)	7 (58%)	13 (54%)	67 (57%)
4D	17 (49%)	18 (38%)	5 (42%)	11 (46%)	51 (43%)
CTV/ITV to PTV expansion margin, median (range), mm					
Superior	9 (5-15)	10 (5-15)	10 (5-15)	10 (5-15)	10 (5-15)
Inferior	9 (5-15)	10 (5-15)	10 (5-15)	10 (5-15)	10 (5-15)
Lateral	5 (5-10)	5 (5-10)	5 (5-10)	5 (5-10)	5 (5-10)
PTV V90%, median (range), %	99.6 (97.0-100.0)	99.9 (93.3-100.0)	100.0 (99.2-100.0)	99.9 (98.5-100.0)	99.9 (93.3-100.0)
CTV V95%, median (range), %	100.0 (97.6-100.0)	100.0 (88.3-100.0)	100.0 (99.1-100.0)	100.0 (94.8-100.0)	100.0 (88.3-100.0)
Intravenous contrast					
Imaged without	16 (46%)	15 (32%)	2 (17%)	4 (17%)	37 (31%)
Imaged with	19 (54%)	32 (68%)	10 (83%)	20 (83%)	81 (69%)

*Abbreviations:* C/I/PTV = clinical/internal/planning target volume; CT = computed tomography; RT = radiation therapy; VMAT = volumetric modulated arc therapy.

∗ Of 120 patients recruited, 118 began treatment.

† Including first and last day of RT.

**Fig. 1:**
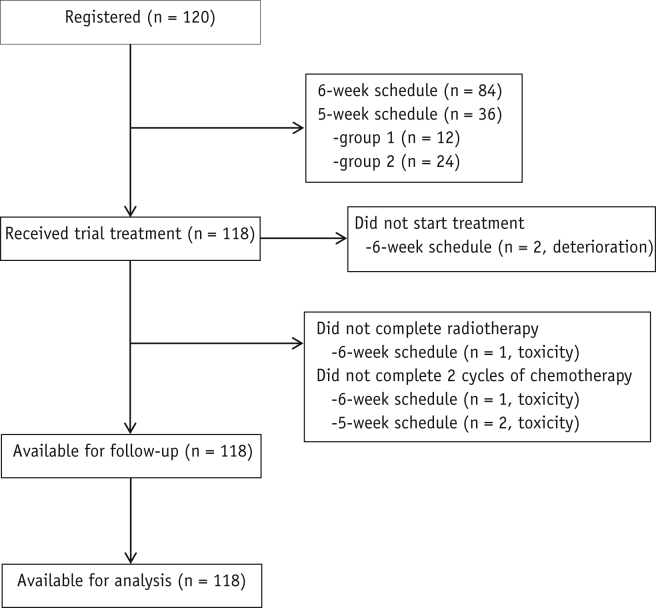
CONSORT diagram.

A CONSORT diagram is provided in Figure 1. Of the 84 6-week patients, 2 did not start treatment owing to deterioration, and 1 completed only 1 cycle of chemotherapy and did not finish RT owing to toxicity. All 36 5-week patients completed RT, but because of toxicity 2 patients missed 1 cycle of chemotherapy and another 2 missed 1 dose within a chemotherapy cycle. The median prescribed dose was 64.5 Gy (range, 63.0-71.0 Gy) for 5-week patients, 67.6 Gy (range, 63.0-73.0 Gy) for treated 6-week patients, and 66.0 Gy (range, 63.0-73.0 Gy) overall.

### Toxicity

Incidences of radiation pneumonitis and early esophagitis are summarized in Table 3 (n = 118). Grade ≥2 pneumonitis was seen in 25.0% (95% CI, 12.1-42.2%) of all 5-week patients (n = 36), and in 20.8% (95% CI, 7.1-42.2%) of Group 2 5-week patients (n = 24), with 1 grade 3 case in Group 2 (4.2%; upper one-sided 90% CI, 15.3%; below the prespecified unacceptable rate of 20%). For the 6-week schedule (n = 82), grade ≥2 pneumonitis occurred in 30.5% (95% CI, 20.8-41.6%) of patients, with 3 grade 3 cases (3.7%; 95% CI, 0.8-10.3%). Across both schedules the grade 3 pneumonitis rate was 3.4% (95% CI, 0.9-8.5%).

**Table 3:** Incidence of pneumonitis, esophagitis, adverse events, and adverse events at least possibly related to radiation therapy∗

Toxicity and grade	6-week schedule		5-week schedule		Total
	Group 1 (n = 35)	Group 2 (n = 47)	Group 1 (n = 12)	Group 2 (n = 24)	(n = 118†)
Pneumonitis					
1	13 (37%)	11 (23%)	1 (8%)	4 (17%)	29 (25%)
2	10 (29%)	12 (26%)	4 (33%)	4 (17%)	30 (25%)
3	2 (6%)	1 (2%)	0	1 (4%)	4 (3%)
4	0	0	0	0	0
5	0	0	0	0	0
Esophagitis					
1	1 (3%)	4 (9%)	0	0	5 (4%)
2	30 (86%)	33 (70%)	11 (92%)	22 (92%)	96 (81%)
3	2 (6%)	3 (6%)	1 (8%)	1 (4%)	7 (6%)
4	0	0	0	0	0
5	0	0	0	0	0
Any adverse event					
1	0	0	0	0	0
2	8 (23%)	10 (21%)	5 (42%)	6 (25%)	29 (25%)
3	21 (60%)	26 (55%)	7 (58%)	18 (75%)	72 (61%)
4	4 (11%)	9 (19%)	0	0	13 (11%)
5	2 (6%)	2 (4%)	0	0	4 (3%)
Any adverse event at least possibly related to radiation therapy					
1	0	2 (4%)	0	0	2 (2%)
2	17 (49%)	19 (40%)	8 (67%)	17 (71%)	61 (52%)
3	13 (37%)	20 (43%)	4 (33%)	7 (29%)	44 (37%)
4	3 (9%)	6 (13%)	0	0	9 (8%)
5	2 (6%)	0	0	0	2 (2%)

∗ The table summarizes the highest grades of pneumonitis, esophagitis, and adverse events experienced by each patient.

† Of 120 patients recruited, 118 began treatment.

Grade 3 early esophagitis was seen in 5.6% (95% CI, 0.7%-18.7%) of 5-week patients, 6.1% (95% CI, 2.0%-13.7%) of 6-week patients, and 5.9% (95% CI, 2.4%-11.8%) overall. Late grade 2 esophageal toxicities (>3 months after the start of RT) occurred in 11.1% (95% CI, 3.1%-26.1%) of 5-week patients, including 25.0% of patients in Group 2 (95% CI, 5.5%-57.2%), and in 17.1% (95% CI, 9.7%-27.0%) of 6-week patients. One 6-week patient who received 71 Gy to 1 cm^3^ of esophagus experienced a grade 5 perforation.^7^

Grade ≥3 AEs considered at least possibly RT-related occurred in 11 of the 5-week patients (30.6%; 95% CI, 16.3%-48.1%) and 44 of the 6-week patients (53.7%; 95% CI, 42.3%-64.7%), as detailed in Table 3 and Table E2 (available online at https://doi.org/10.1016/j.ijrobp.2019.11.397). Grade ≥4 AEs were seen in no 5-week patients versus 20.7% (95% CI, 12.6%-31.1%) of 6-week patients, a significant difference (*P* = .03, allowing for the 6-week patients’ longer follow-up). Of 4 grade 5 AEs, 2 were considered RT related (Table 3).^7^

### Tumor response and survival

At 3 months post-RT, 44.4% and 63.4% of patients treated using the 5- and 6-week schedules had a partial response, 19.4% and 25.6% stable disease, 27.8% and 4.9% progressive disease, 5.6% and 4.9% were unevaluable, and one 5-week patient (2.8%) and one 6-week patient (1.2%) had died.

**Fig. 2:**
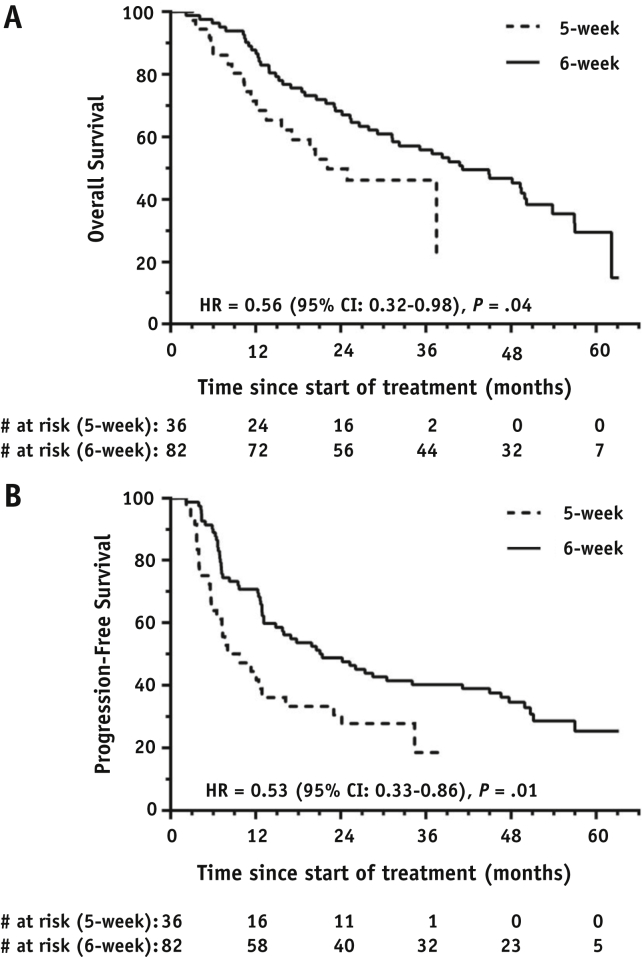
Kaplan-Meier curves of (A) overall survival and (B) progression-free survival.

OS and PFS are detailed in Table 4. In 6-week patients, after a median follow-up of 51.8 months, 25 were alive and progression free, 5 were alive having progressed, and 52 had died; median OS and PFS were 41.2 months (95% CI, 28.5-53.9) and 21.1 months (95% CI, 10.9-31.2), respectively. In 5-week patients, after a median follow-up of 26.4 months, 9 were alive and progression free, 8 were alive having progressed, and 19 had died; median OS and PFS were 22.1 months (95% CI, 12.9-31.3) and 8.0 months (95% CI, 2.2-13.9). Survival differed significantly between 6-week and 5-week patients (Fig. 2), with HRs of 0.56 (95% CI, 0.32-0.98; *P* = .04) for OS and 0.53 (95% CI, 0.33-0.86; *P* = .01) for PFS. Across both schedules median OS and PFS were 37.5 months (95% CI, 26.1-48.8) and 16.0 months (95% CI, 8.7-23.2) after a median follow-up of 50.0 months.

**Table 4:** Overall and progression-free survival

	6-week schedule (n = 82)	5-week schedule (n = 36)	Total (n = 118∗)
Median follow-up, mo	51.8	26.4	50.0
*OS*			
Events, deaths	52	19	71
1-year OS (95% CI), %	87.8 (80.7-94.9)	71.4 (56.3-86.5)	82.9 (76.0-89.8)
2-year OS (95% CI), %	68.3 (58.3-78.3)	49.8 (32.7-66.9)	62.9 (54.1-71.7)
Median OS (95% CI), mo	41.2 (28.5-53.9)	22.1 (12.9-31.3)	37.5 (26.1-48.8)
6- vs 5-week HR (95% CI)	0.56 (0.32-0.98)		
*P* value	.04		
*PFS*			
Events, progressions/deaths	57	27	84
1-year PFS (95% CI), %	70.7 (60.9-80.5)	44.4 (28.1-60.7)	62.7 (53.9-71.5)
2-year PFS (95% CI), %	48.8 (38.0-59.6)	30.6 (15.5-45.7)	43.2 (34.2-52.2)
Median PFS (95% CI), mo	21.1 (10.9-31.2)	8.0 (2.2-13.9)	16.0 (8.7-23.2)
6- vs 5-week HR (95% CI)	0.53 (0.33-0.86)		
*P* value	.01		

*Abbreviations:* CI = confidence interval; HR = hazard ratio; OS = overall survival; PFS = progression-free survival.

∗ Of 120 patients recruited, 118 began treatment.

Associations between OS and patient- and treatment-related factors are summarized in Table 5. In univariable analyses, OS was significantly associated with treatment schedule and CTV/ITV V_95%_, the fractional volume of CTV (for 3D-CT imaged patients) or ITV (4D-CT) receiving ≥95% of the prescribed dose level (HR, 0.88; 95% CI, 0.77-1.00; *P* = .05). In a multivariable model including these 2 factors, similar associations with OS remained (*P* = .02 for schedule, *P* = .03 for CTV/ITV V_95%_), and there was no evidence of an interaction effect (*P* = .94).

**Table 5:** Associations between overall survival and patient- and treatment-related factors

Factor	Unadjusted HR (95% CI); *P* value	Adjusting for schedule HR (95% CI); *P* value
*Patient related*		
Age, y	1.02 (0.99-1.05); *P* = .13	1.02 (0.99-1.06); *P* = .12
Sex (male vs female)	1.18 (0.70-1.98); *P* = .53	1.29 (0.76-2.18); *P* = .34
Stage (≥IIIB vs <IIIB)	1.13 (0.67-1.91); *P* = .65	1.10 (0.65-1.87); *P* = .71
WHO PS (1 vs 0)	1.18 (0.73-1.92); *P* = .49	1.28 (0.79-2.09); *P* = .32
GTV absolute volume (cm^3^)	1.00 (1.00-1.00); *P* = .54	1.00 (1.00-1.00); *P* = .42
*Treatment related*		
Schedule (6- vs 5-wk)	0.56 (0.32-0.98); *P* = .04	N/A∗
Prescribed dose (Gy)	0.95 (0.89-1.02); *P* = .16	0.95 (0.89-1.02); *P* = .16
Technique (VMAT vs 3D conformal)	1.50 (0.74-3.04); *P* = .26	1.17 (0.55-2.50); *P* = .69
CT (4D vs 3D)	0.79 (0.49-1.27); *P* = .32	0.79 (0.49-1.27); *P* = .32
PTV V_90%_	0.91 (0.73-1.14); *P* = .42	0.87 (0.70-1.08); *P* = .22
CTV/ITV V_95%_ (%)	0.88 (0.77-1.00); *P* = .05	0.86 (0.76-0.99); *P* = .03
Heart V_20Gy_ (%)	1.00 (0.99-1.02); *P* = .94	1.00 (0.99-1.02); *P* = .67
Heart V_40Gy_ (%)	1.01 (0.99-1.04); *P* = .31	1.02 (0.99-1.05); *P* = .13
Heart V_60Gy_ (%)	1.03 (0.97-1.08); *P* = .37	1.04 (0.98-1.10); *P* = .19
Heart mean dose (Gy)	1.00 (0.97-1.04); *P* = .87	1.01 (0.98-1.04); *P* = .56
Lung V_20Gy_ (%)	1.01 (0.97-1.05); *P* = .64	1.01 (0.97-1.04); *P* = .68
Lung V_40Gy_ (%)	0.99 (0.94-1.05); *P* = .81	1.00 (0.95-1.05); *P* = .91
Lung V_60Gy_ (%)	1.03 (0.93-1.15); *P* = .53	1.05 (0.95-1.17); *P* = .32
Lung mean dose (Gy)	1.01 (0.93-1.10); *P* = .80	1.02 (0.94-1.10); *P* = .67
CTV/ITV to PTV expansion margin		
Superior (mm)	1.05 (0.96-1.14); *P* = .33	1.04 (0.95-1.14); *P* = .38
Inferior (mm)	1.04 (0.96-1.14); *P* = .35	1.04 (0.95-1.14); *P* = .40
Lateral (mm)	0.97 (0.87-1.08); *P* = .58	0.98 (0.88-1.09); *P* = .68

*Abbreviations:* CI = confidence interval; CTV = clinical target volume; GTV = gross tumor volume; HR = hazard ratio; PTV = planning target volume; VMAT = volumetric modulated arc therapy; WHO-PS = World Health Organization performance status.

∗ The effect size for treatment schedule (6- vs 5-week) remained similar after adjusting for each of the other patient- and treatment-related factors individually and remained statistically significant except after adjusting for prescribed dose (adjusted HR, 0.60; 95% CI, 0.33-1.08; *P* = .09) or technique (adjusted HR, 0.58; 95% CI, 0.32-1.06; *P* = .08) when weak evidence of a difference still remained.

Patient age and prescribed dose also had *P* values <.20 in univariable analyses, and when these quantities were additionally considered in multivariable analyses, the model with the best Akaike Information Criterion score comprised 3 factors: treatment schedule (adjusted HR, 0.51; 95% CI, 0.29-0.91; *P* = .02), CTV/ITV V_95%_ (adjusted HR, 0.86; 95% CI, 0.75-0.99; *P* = .03), and age (adjusted HR, 1.02; 95% CI, 0.99-1.06; *P* = .13).

## Discussion

IDEAL-CRT trialed an individualized, moderately dose-per-fraction escalated concurrent CRT treatment of NSCLC in a multicenter setting and demonstrated feasibility, acceptable toxicity, and promising efficacy when delivered over 6 weeks. Of 118 patients who began treatment, 113 (96%) completed it as planned, and 2 (2%) missed only 1 dose of a chemotherapy cycle.

At 3.4%, the overall grade ≥3 radiation pneumonitis rate in IDEAL-CRT was slightly lower than in either arm of RTOG-0617^4^ or the average rate obtained from meta-analysis of concurrent CRT results.^16^ The overall grade ≥3 esophagitis rate in IDEAL-CRT was 5.9%, lower than rates in either arm of RTOG-0617 or the MAASTRO study of isotoxically dose-escalated concurrent CRT,^8^ or average rates in 2 meta-analyses of concurrent CRT.^2^^,^^16^

One IDEAL-CRT patient from the 6-week Group 1 cohort receiving 71 Gy to 1 cm^3^ of esophagus experienced a fatal esophageal perforation. No other late grade ≥3 esophageal toxicity was seen, and the esophageal maximum tolerated dose for the 6-week schedule was defined as 68 Gy.^7^ For the 5-week schedule, toxicity was acceptable for the highest esophageal dose limit trialed, 65 Gy. The overall rate of severe esophageal toxicity was lower in IDEAL-CRT than in the RTOG-0617 or MAASTRO dose-escalation studies,^4^^,^^8^ or in a Netherlands Cancer Institute trial of concurrent CRT.^17^

There were 2 treatment-related deaths in IDEAL-CRT (1.7%), similar to the rate in RTOG-0617 (3.3%) and the average rate for concurrent CRT in a meta-analysis (3.8%).^16^ Overall, toxicity in IDEAL-CRT appears no higher than in comparable studies of concurrent CRT.

Survival in IDEAL-CRT was promising, with a median OS of 37.5 months across all patients treated. For 6-week patients, median OS was 41.2 months, comparing well with 28.7 months in the RTOG-0617 baseline arm, which also delivered 30 fractions in 40 days and set a new benchmark for survival in patients with locally advanced NSCLC.^4^ Caution should be exercised when comparing results in phase 1/2 and phase 3 studies, but it is notable that survival for the experimental arm of RTOG 0617 was similar in phase 1/2 and phase 3 (median OS of 21.6 and 20.3 months, respectively, for stage III patients).^4^^,^^18^

The IDEAL-CRT 6-week median prescribed dose of 67.6 Gy represents a 9% increase in tumor EQD2 beyond the RTOG-0617 baseline isocenter dose (for 10 Gy α/β^9^), which may have contributed to the longer survival seen in IDEAL-CRT 6-week patients. Median fractional heart volumes receiving ≥5 and 30 Gy were 39.6% and 10.3% for IDEAL-CRT 6-week patients versus 50.4% and 14.3% for the RTOG-0617 baseline arm. The smaller irradiated volumes in IDEAL-CRT probably reflect the relatively tight tumor margins used (see Methods and Materials) and may have contributed to the longer survival, because cardiac irradiation has been found to be negatively associated with survival in some studies.^4^^,^^19^

IDEAL-CRT 6-week patients were more likely to be older and male than RTOG-0617 patients, with squamous rather than adenocarcinoma histology and worse PS, all factors associated with poorer OS.^20^, ^21^, ^22^ Thus, their longer survival is not due to these factors. Fewer IDEAL-CRT 6-week patients had stage IIIB disease (25% vs 34% for RTOG-0617) and their tumors were slightly smaller (median GTV 108 cm^3^ vs 123.1 cm^3^ for RTOG-0617), but within the IDEAL-CRT data set these factors were not significantly associated with OS.

For 5-week IDEAL-CRT patients, median OS was 22.1 months, significantly shorter than for 6-week patients. Although patients were not randomized between the 2 schedules, this survival difference is unlikely to be due to patient factors because sex, disease stage, PS, and GTV size were not associated with OS in the IDEAL-CRT data set. Increasing age was marginally negatively associated with OS (*P* = .13), but median age was slightly lower for 5- than for 6-week patients. Nor is the survival difference center related, since all but 2 of the 5-week patients were recruited from the same centers as the 6-week patients. Furthermore, results were consistent in sensitivity analyses when the 2 patients from the additional 5-week center were excluded (HR, 0.56; 95% CI, 0.32-0.99; *P* = .05).

The median prescribed dose was 3.1 Gy lower for 5- than for 6-week patients. We had expected the effect of this dose reduction to be negated or reversed by the decreased scope for tumor repopulation provided by the shorter schedule,^9^ but it is now thought that concurrent chemotherapy may substantially limit repopulation.^23^ The shorter survival of 5-week patients might, therefore, result simply from the slightly lower doses they receive. However, prescribed dose was not significantly associated with OS in IDEAL-CRT, whereas schedule was more significantly associated with OS than with any other factor. The longer survival of 6-week IDEAL-CRT patients was accompanied by a significantly higher rate of grade ≥4 AEs, suggesting that the 6-week schedule had more effect on both tumors and normal tissues. We are currently analyzing IDEAL-CRT follow-up CT scans to understand the etiology of late radiation-induced lung damage.^24^^,^^25^

CTV/ITV V95% was significantly associated with OS in IDEAL-CRT, with an HR of 0.88 representing a 12% increase in the hazard rate for death per 1% loss in CTV/ITV fractional volume receiving ≥95% of the prescribed dose. When patients were dichotomized into those with CTV/ITV V_95%_ >99% or ≤99% (105 vs 11 patients), an HR of 0.46 (95% CI, 0.23-0.90) was found, favoring the V_95%_ >99% group. But when V_95%_ was analyzed as a continuous variable for the V_95%_ >99% group alone, an HR of 0.99 (95% CI, 0.37-2.64) was observed, indicating little variation in survival with CTV/ITV V_95%_ when coverage was better than 99%.

Fractional heart volumes receiving ≥20, 40, and 60 Gy were not associated with OS (Table 5). A more detailed principal component–based analysis carried out for 6-week IDEAL-CRT patients has found a significant negative association between OS and heart volumes receiving 63 to 69 Gy,^19^ and a similar analysis is underway for the combined 5- and 6-week data set.

The recent PACIFIC phase 3 trial of 54 to 66 Gy concurrent CRT followed either by the immune agent durvalumab or a placebo has reported significantly longer survival for the durvalumab arm,^26^^,^^27^ raising the possibility that the encouraging IDEAL-CRT 6-week survival might be further lengthened by following the treatment with an immune agent.

## Conclusions

IDEAL-CRT trialed individualized, moderately dose-escalated concurrent CRT, given in 5- and 6-week schedules, and found good patient compliance and acceptable toxicity. Survival for patients treated using the 6-week schedule was promising and in this nonrandomized study was significantly and substantially longer than for 5-week patients, who received a 3.1 Gy lower median dose. The rate of high-grade AEs was also greater in 6-week patients. Survival was shorter in patients with ≤99% coverage of the CTV/ITV by 95% of the prescribed dose. The encouraging survival seen for patients treated using the 6-week schedule might be further improved by following it with an immune agent, motivating further study of such combined optimized treatments.

## Supplementary Data

Tables E1 and E2
