# Supplementary material for: Long-Term Results from the IDEAL-CRT Phase 1/2 Trial of Isotoxically Dose-Escalated Radiation Therapy and Concurrent Chemotherapy for Stage II/III Non-small Cell Lung Cancer
Source: Int J Radiat Oncol Biol Phys. 2020 Mar 15;106(4):733–42. doi: 10.1016/j.ijrobp.2019.11.397 (PMC7049901; doi:10.1016/j.ijrobp.2019.11.397)
Supplement: Tables E1 and E2 [file mmc1.pdf]

Supplementary Table 1. IDEAL-CRT isotoxic dose-prescription.

Dose-prescription	To ICRU reference-point.
Target coverage	≥ 98% of PTV covered by ≥ 90% prescribed dose.
Initial dose-prescription based on lung-GTV	Prescribed tumor dose initially chosen so that lung-GTV $EQD2_{mean}$ was 18.2 Gy, then reduced by 10% to allow for possible intensification of radiation effect by concurrent chemotherapy.
Maximum tumor prescribed dose	73 and 71 Gy for the 6- and 5-week schedules. Higher initial prescribed doses were reduced to these levels.
Minimum tumor prescribed dose	63 Gy. Lower initial prescribed doses were raised to this level provided the resulting lung-GTV $EQD2_{mean}$ was ≤ 19.3 Gy and V_{20}† ≤ 35%. Otherwise the patient was ineligible for IDEAL-CRT.
Other normal tissue dose-constraints	Tumor prescribed doses were reduced further if necessary to meet the normal tissue dose-limits below. A resulting prescribed dose <63 Gy made a patient ineligible for IDEAL-CRT.
Spinal Cord	$D_{0.1cc}$* ≤ 47Gy
Brachial plexus	$D_{30\%}$ ≤ 60Gy, $D_{0.1cc}$ ≤ 65Gy
Heart	$D_{100\%}$ < 45 Gy, $D_{67\%}$ < 53 Gy, $D_{33\%}$ < 60 Gy (6-week schedule)<br>$D_{100\%}$ < 44 Gy, $D_{67\%}$ < 52 Gy, $D_{33\%}$ < 59 Gy (5-week schedule)
Esophagus	Dose to 1 cc = 65 Gy for Group 1 (6- & 5-week)<br>Dose to 1 cc = 68 Gy for Group 1 (6-week only)<br>Dose to 1 cc = 71 Gy for Group 1 (6-week only)<br>Dose to 1 cc ≤ 63 Gy** for Group 2 (6- & 5-week)

† V_X denotes the percentage of a structure receiving more than X Gy.

* $D_{X[cc\ or\ \%]}$ denotes the minimum dose delivered to the most highly irradiated X cc or X% of the tissue.

** For the 6 week schedule this dose-level was increased to 65 Gy, and then 68 Gy as safety data became available from Group 1.

Supplementary Table 2. Grade ≥3 adverse events, and grade ≥3 adverse events at least possibly radiotherapy-related, listed for events with rates ≥5%.

	6-week schedule (N=82)	5-week schedule (N=36)	Total (N=118)
**Any adverse event**			
Lung infection	20 (24.4%)	5 (13.9%)	25 (21.2%)
Decreased FEV	14 (17.1%)	2 (5.6%)	16 (13.6%)
Decreased neutrophils	12 (14.6%)	0 (0.0%)	12 (10.2%)
Decreased WBC	11 (13.4%)	1 (2.8%)	12 (10.2%)
Dyspnoea	12 (14.6%)	0 (0.0%)	12 (10.2%)
Decreased lymphocytes	9 (11.0)	2 (5.6%)	11 (9.3%)
Pulmonary embolism	7 (8.5%)	2 (5.6%)	9 (7.6%)
Decreased CO diffusing capacity	6 (7.3%)	2 (5.6%)	8 (6.8%)
Fatigue	7 (8.5%)	0 (0.0%)	7 (5.9%)
Late radiation fibrosis	6 (7.3%)	1 (2.8%)	7 (5.9%)
Esophagitis	5 (6.1%)	2 (5.6%)	7 (5.9%)
Nausea	5 (6.1%)	1 (2.8%)	6 (5.1%)
**Any adverse event at least possibly radiotherapy-related**			
Decreased FEV	12 (14.6%)	2 (5.6%)	14 (11.9%)
Lung infection	8 (9.8%)	2 (5.6%)	10 (8.5%)
Decreased neutrophils	7 (8.5%)	0 (0.0%)	7 (5.9%)
Decreased CO diffusing capacity	6 (7.3%)	1 (2.8%)	7 (5.9%)
Decreased WBC	7 (8.5%)	0 (0.0%)	7 (5.9%)
Late radiation fibrosis	6 (7.3%)	1 (2.8%)	7 (5.9%)
Esophagitis	5 (6.1%)	2 (5.6%)	7 (5.9%)
